# A survey of the use and impact of *International Journal of Epidemiology*'s Education Corner

**DOI:** 10.1093/ije/dyac160

**Published:** 2022-08-24

**Authors:** Ellie Medcalf, Jonathan Y Huang, Onyebuchi A Arah, Michael O Harhay, Stephen R Leeder, Katy J L Bell

**Affiliations:** School of Public Health, Faculty of Medicine and Health, University of Sydney, Sydney, NSW, Australia; Editorial Board, International Journal of Epidemiology, Sydney, Australia; Biostatistics and Human Development, Singapore Institute for Clinical Sciences, Agency for Science, Technology and Research, Singapore, Singapore; Centre for Quantitative Medicine, Duke-NUS Medical School, Singapore, Singapore; Editorial Board, International Journal of Epidemiology, Sydney, Australia; Department of Epidemiology, Fielding School of Public Health, University of California Los Angeles, Los Angeles, CA, USA; Department of Statistics, UCLA College, Los Angeles, CA, USA; Department of Public Health, Research Unit for Epidemiology, Aarhus University, Aarhus, Denmark; Editorial Board, International Journal of Epidemiology, Sydney, Australia; Department of Biostatistics, Epidemiology and Informatics, Perelman School of Medicine, University of Pennsylvania, Philadelphia, PA, USA; Clinical Trials Methods and Outcomes Lab, Palliative and Advanced Illness Research Center, Perelman School of Medicine, University of Pennsylvania, Philadelphia, PA, USA; School of Public Health, Faculty of Medicine and Health, University of Sydney, Sydney, NSW, Australia; Editor-in-Chief, International Journal of Epidemiology, Sydney, Australia; School of Public Health, Faculty of Medicine and Health, University of Sydney, Sydney, NSW, Australia; Editorial Board, International Journal of Epidemiology, Sydney, Australia

The *International Journal of Epidemiology* (*IJE*) Education Corner was introduced in 2012,^1^ providing educational articles on epidemiological concepts and methods. We conducted a survey of the use and impact of the *IJE* Education Corner articles in relation to teaching, research and practice, collating suggestions on how we might increase their utility.

Questions for the Survey were developed iteratively by the Education Corner editors and *IJE* editorial staff. A 29-item online survey was administered between 11 November 2021 and 14 January 2022, using mailing lists of the *International Journal of Epidemiology* editorial board, the International Epidemiology Association and the World Congress of Epidemiology. The survey link was also posted on Twitter using @IntJEpidemiol and the hashtags #epitwitter #statstwitter. Study data were collected and managed using REDCap electronic data capture tools^2^^,^^3^ hosted at the University of Sydney. We used mixed methods to analyse the results, with descriptive statistics for quantitative data and content analysis for qualitative data including free-text answers.^4^

We received 213 responses from an estimated 2000 people invited to undertake the survey (denominator estimated from e-mail lists, does not include invitation via twitter; response rate approximately 11%). Of respondents, 56% (120) self-identified as male, 43% (92) as female and 1% (1) as other. Respondents were from 54 countries and their mean age was 46.4 years. Most had a PhD, DPhil, DSc or ScD in public health or epidemiology (64%) and worked in an academic role in teaching and/or research (82%) (Table 1).

**Table 1:** *IJE* Education Corner: characteristics of survey respondents

Characteristic	Measure
Age, years	
Median (IQR)	44 (17)
Min, max	21, 81
Sex, *n* (%)	
Male	120 (56%)
Female	92 (43%)
Other	1 (1%)
Country, *n* (%)	
Australia	59 (28%)
India	23 (11%)
USA	20 (9%)
Nigeria	11 (5%)
UK	11 (5%)
Brazil	6 (3%)
Japan	6 (3%)
China	5 (2%)
Mexico	5 (2%)
Cameroon	4 (2%)
Canada	4 (2%)
Germany	4 (2%)
Austria	3 (1%)
Colombia	3 (1%)
Lebanon	3 (1%)
Other^a^	46 (22%)
Regions, *n* (%)	
Oceania	60 (28%)
Americas	44 (21%)
North West Europe	27 (13%)
Southern and Central Asia	26 (12%)
Sub-Saharan Africa	20 (9%)
North Africa and Middle East	13 (6%)
North East Asia	12 (6%)
Southern and Eastern Europe	6 (3%)
South East Asia	5 (2%)
Source of invitation, *n* (%)	
International Epidemiology Association	94 (44%)
*International Journal of Epidemiology* Editorial Board	55 (26%)
World Congress of Epidemiology	47 (22%)
Epi Twitter	12 (6%)
Other^b^	5 (2%)
Qualifications, *n* (%)	
PhD/DPhil/DSc/ScD in public health/epidemiology	136 (64%)
Master’s degree in public health/epidemiology	92 (43%)
Medical degree (e.g. MBBS, MBChB, MD)	69 (32%)
Undergraduate degree in public health/epidemiology	18 (9%)
Area of work, *n* (%)	
Academic (research/teaching)	175 (82%)
Public health practice	58 (27%)
Clinical practice	20 (9%)
Health service administration	13 (6%)
Other^c^	8 (4%)

IQR, interquartile range; min, max minimum and maximum.

a There were two respondents from Bangladesh, Denmark, Iran, Morocco, Norway, Peru and Singapore, and one respondent from Bahrain, Chile, Cuba, Czech Republic, Egypt, Ethiopia, Finland, France, Italy, Kuwait, Latvia, Myanmar, The Netherlands, New Zealand, Oman, Panama, Portugal, Russia, Rwanda, South Africa, South Korea, Spain, Sri Lanka, Sudan, Sweden, Switzerland, Thailand, Togo, Tunisia, Uganda, United Arab Emirates and Uruguay.

b Three answered Asociación Mexicana de Inteligencia Epidemiológica, one answered The Epidemiological Monitor and one answered e-mail.

c Each of the following responses were provided once: Agriculture industry/anthropology/qualitative health/ATLAS.ti, clinical research, contract researcher, epidemiology-related surveys, not-for-profit, PhD student, regulatory affairs, social epidemiology.

Of the 213 respondents, 43% (91) said that they had used *IJE* Education Corner articles in their teaching, research or practice: 24% (52) used Education Corner articles specifically in their teaching (45 in epidemiology courses), 33% (70) in their research and 10% (22) in their public health/clinical practice (Table 2). We also found that Education Corner articles are used in teaching, research and practice across all regions of the world (Table 2).

**Table 2:** Use of Education Corner articles in teaching, research and practice

	Teaching, *n* (%)	Research, *n* (%)	Practice, *n* (%)
Number of respondents who used the articles in teaching or research	52	70	22
Geographical region			
Oceania	6 (12%)	17 (24%)	3 (14%)
Americas	16 (31%)	19 (27%)	5 (23%)
North West Europe	6 (12%)	9 (13%)	1 (5%)
Southern and Central Asia	4 (8%)	6 (9%)	6 (27%)
Sub-Saharan Africa	5 (10%)	6 (9%)	3 (14%)
North Africa and Middle East	7 (13%)	4 (6%)	3 (14%)
North East Asia	4 (8%)	7 (10%)	1 (5%)
Southern and Eastern Europe	4 (8%)	2 (3%)	–
South East Asia	–	–	–
Course in which Education Corner articles were used^a^ (n = 52)			
Epidemiology	45 (87%)	–	–
Biostatistics	15 (29%)	–	–
Other	4 (8%)	–	–
Course level^b^ (*n *= 52)		–	–
Beginner	17 (33%)	–	–
Intermediate	26 (50%)	–	–
Advanced	24 (46%)	–	–
Number of Education Corner articles used (*n* = 122)^c^		–	–
Median (IQR)	2 (2)	2 (2)	–
1	15 (29%)	19 (27%)	–
2	15 (29%)	20 (29%)	–
3	10 (19%)	10 (14%)	–
4	3 (6%)	2 (3%)	–
5	4 (8%)	4 (6%)	–
10		1 (1%)	–

IQR, interquartile range.

a Eleven respondents answered ‘Yes’ to both epidemiology and biostatistics courses, two answered ‘Yes’ to both epidemiology and other courses.

b Three respondents answered ‘Yes’ to using in all of beginners, intermediate and advanced courses; four answered ‘Yes’ to using in both beginners and intermediate courses; six answered ‘Yes’ to using in both intermediate and advanced courses.

c Data missing for five respondents who used articles in their teaching and 14 respondents who used articles in their research.

Many respondents had used at least two Education Corner articles in their teaching or research, with basic epidemiological study designs the most popular topic. For example, the top two articles selected by respondents for use in teaching and research were ‘Classification of epidemiological study designs’^5^ and ‘Case-control studies: basic concepts’.^6^ Other popular articles included introductions to epidemiological concepts and methods, such as ‘Mediation analysis in epidemiology: methods, interpretation and bias’^7^ and ‘Competing risks in epidemiology: possibilities and pitfalls’.^8^ These and other articles that survey respondents reported using are shown in Supplementary Table S1 (available as Supplementary data at *IJE* online).

Respondents who did not use *IJE* Education Corner articles in their teaching, research or practice provided suggestions for increasing their engagement with articles (Supplementary Table S2, available as Supplementary data at *IJE* online). Many expressed their lack of awareness of *IJE* Education Corner articles but a willingness to use them in the future.

‘I was not aware of this resource … I may use them in the future now that I am aware.’

‘I don’t teach classes, but plan to check it out to help support HDR [higher degree by research] students. I just didn’t know it existed.’

Given this lack of familiarity with the Education Corner, respondents also suggested that there was a need for increased promotion and accessibility of Education Corner articles.

‘E-mail reminders of the release of issues, with a table of contents, may motivate me to read the Education Corner and use it in my teaching.’

‘An index of articles so I can easily identify them.’

‘I think it needs to be better publicised on the webpage and in social media.’

Respondents also indicated that they would be more likely to use Education Corner articles if topics were relevant to their work. Some indicated that papers covering foundational epidemiology would be most useful to them, whereas others wanted papers covering more advanced epidemiological concepts, or specific topics more applicable to their area of work.

‘I think the articles are very good, but I mostly teach undergraduate medical students, so the articles are too advanced.’

‘Some more basic concepts would be good for foundation-level students.’

‘Wider scope from basic to advanced.’

‘Educational topics of comprehensive and state-of-the-art subjects.’

‘Case studies for teaching epidemiological research from diverse contexts, especially community settings.’

‘Methodology on environmental epidemiology, especially climate change and health research.’

‘Free [Education Corner] full texts from research in developing countries.’

These survey data have formed part of an evaluation of Education Corner by a team of *IJE* editors, and inform the expanded vision for the Education Corner, outlined in the accompanying Editorial.^9^ In particular, we have changed the format in the hope of increasing accessibility and encouraging uptake of both basic and advanced methods. We will also be increasing outreach to under-represented voices at all career stages. We plan to continue to grow Education Corner for those learning about and using epidemiology wherever they are in the world, as a contribution to the health of populations.

## Ethics approval

This negligible risk study was assessed by the University of Sydney Human Research Ethics Office and exempt from formal ethical review.

## Data availability

Available on request.

## Supplementary data

Supplementary data are available at *IJE* online.

## Author contributions

E.M. did the data analysis, contributed to data interpretation and wrote the original draft of the manuscript. J.H., O.A., M.H. and S.L. contributed to conceptualization, recruitment and data interpretation, and reviewed and edited the manuscript. K.B. directed the study and was primary supervisor, contriubuted to conceptualization, recruitment and data interpretation, and reviewed and edited the manuscript. Katy Bell is guarantor.

## Funding

There was no specific funding for the study. E.M. and K.B. are supported by an Australian National Health and Medical Research Council (NHMRC) Investigator Grant (#1174523, CIA Bell).

## Supplementary Material

dyac160_Supplementary_Data
