# Supplementary material for: A survey of the use and impact of International Journal of Epidemiology's Education Corner
Source: Int J Epidemiol. 2022 Aug 24;51(5):1687–90. doi: 10.1093/ije/dyac160 (PMC9557904; doi:10.1093/ije/dyac160)
Supplement: dyac160_Supplementary_Data [file dyac160_supplementary_data.docx]

**Supplementary Table S1. Articles most commonly used in teaching or research**

| **Articles used in teaching** | | **Articles used in research** | |
| --- | --- | --- | --- |
| ***Article*** | ***Number who used it*** | ***Article*** | ***Number who used it*** |
| Classification of epidemiological study designs 2012(1) | 17 | Classification of epidemiological study designs 2012(1) | 12 |
| Case-control studies: basic concepts, 2012(2) | 16 | Case-control studies: basic concepts, 2012(2) | 9 |
| Incidence rates in dynamic populations, 2012(3) | 6 | Competing risks in epidemiology: methods, interpretation and bias, 2012(5) | 7 |
| Mediation analysis in epidemiology: methods, interpretation and bias, 2013(4) | 6 | Mediation analysis in epidemiology: methods, interpretation and bias, 2013(4) | 6 |
| Competing risks in epidemiology: methods, interpretation and bias, 2012(5) | 4 | Good practices for quantitative bias analysis, 2014(7) | 5 |
| Time series regression studies in environmental epidemiology, 2013(6) | 4 | Interrupted time series regression for the evaluation of public health interventions 2017(14) | 5 |
| Good practices for quantitative bias analysis, 2014(7) | 4 | Fixed effects analysis of repeated measures data 2014 (19) | 4 |
| Selection bias-a review of recent developments, 2018 (8) | 4 | Statistics education beyond “Significance”: novel plain English interpretations 2020 (17) | 4 |
| Standardized mortality ratios, 2013(9) | 3 | Markov Chain Monte Carlo: an introduction for epidemiologists 2013 (35) | 3 |
| Estimating predicted probabilities from logistic regression 2014 (10) | 2 | Time series regression studies in environmental epidemiology 2013 (6) | 3 |
| Mediation misgivings: ambiguous clinical and public health interpretations 2014 (11) | 2 | Bias from conditioning on live birth in pregnancy cohorts 2015 (12) | 3 |
| Bias from conditioning on live birth in pregnancy cohorts 2015 (12) | 2 | Randomized study designs for lifestyle interventions 2015 (21) | 3 |
| Triangulation in aetiological epidemiology (13) | 2 | Triangulation in aetiological epidemiology 2016 (13) | 3 |

**Supplementary Table S2. Suggestions for increasing use of IJE Education Corner articles in teaching, research and practice**

| **Respondents who had not used the articles in their Teaching, Research and Practice and made suggestions for increasing use** | **N (%)** |
| --- | --- |
| **Total** | 78 (100%) |
| **Geographical Location** |  |
| Oceania | 27 (35%) |
| Americas | 16 (21%) |
| North-West Europe | 10 (13%) |
| Southern and Central Asia | 9 (12%) |
| Sub-Saharan Africa | 4 (5%) |
| North Africa and Middle East | 7 (9%) |
| North-East Asia | 2 (3%) |
| Southern and Eastern Europe | 2 (3%) |
| South-East Asia | 1 (1%) |
| **Common themes in Suggestions^a^** |  |
| More relevant topics (e.g., social epi, genetic epi, research methodology, intervention topics, health promotion, environmental epi (esp. climate change), teaching methods, case studies for teaching epi methods in diverse contexts, short and simple articles about methods) | 35 (45%) |
| Increased awareness/accessibility (e.g., reminders, visibility on webpage) / increased accessibility/ease of use (e.g., searchable collection on the IJE website, index of articles, open access, basic to advanced articles) | 45 (58%) |
| ^a^ Two respondents suggested both themes. | |

**References for Supplementary Table S1**

1. Pearce N. Classification of epidemiological study designs. Int J Epidemiol 2012;41(2):393-7.

2. Vandenbroucke JP, Pearce N. Case―control studies: basic concepts. Int J Epidemiol 2012;41(5):1480-9.

3. Vandenbroucke JP, Pearce N. Incidence rates in dynamic populations. Int J Epidemiol 2012;41(5):1472-9.

4. Richiardi L, Bellocco R, Zugna D. Mediation analysis in epidemiology: methods, interpretation and bias. Int J Epidemiol 2013;42(5):1511-9.

5. Andersen PK, Geskus RB, de Witte T, Putter H. Competing risks in epidemiology: possibilities and pitfalls. Int J Epidemiol 2012;41(3):861-70.

6. Bhaskaran K, Gasparrini A, Hajat S, Smeeth L, Ben A. Time series regression studies in environmental epidemiology. Int J Epidemiol 2013;42(4):1187-95

7. Lash TL, Fox MP, Maclehose RF, Maldonado G, McCandless LC, Greenland S. Good practices for quantitative bias analysis. Int J Epidemiol 2014;43(6):1969-85.

8. Infante-Rivard C, Cusson A. Reflection on modern methods: selection bias-a review of recent developments. Int J Epidemiol 2018;47(5):1714-22.

9. Taylor P. Standardized mortality ratios. Int J Epidemiol 2014;42(6):1882-90.

10. Muller CJ, Maclehose RF. Estimating predicted probabilities from logistic regression: different methods correspond to different target populations. Int J Epidemiol 2014;43(3):962-70.

11. Naimi AI, Kaufman JS, Maclehose RF. Mediation misgivings: ambiguous clinical and public health interpretations of natural direct and indirect effects. Int J Epidemiol 2014;43(5):1656-61.

12. Liew Z, Olsen J, Cui X, Ritz B, Arah OA. Bias from conditioning on live-births in pregnancy cohorts: an illustration based on neurodevelopment in children after prenatal exposure to organic pollutants. Int J Epidemiol 2015;44(1):345-54.

13. Lawlor DA, Tilling K, Davey Smith G. Triangulation in aetiological epidemiology. Int J Epidemiol 2016;45(6):1866-86.

14. Bernal JL, Cummins S, Gasparrini A. Interrupted time series regression for the evaluation of public health interventions: a tutorial. Int J Epidemiol 2017;46(1):348-55.

15. Naimi AI, Cole SR, Kennedy EH. An introduction to g methods. Int J Epidemiol 2017;46(2):756-62.

16. Piel FB, Fecht D, Hodgson S, et al. Small-area methods for investigation of environment and health. Int J Epidemiol 2020;49(2):686-99.

17. Watt HC. Reflection on modern methods: Statistics education beyond 'significance': novel plain English interpretations to deepen understanding of statistics and to steer away from misinterpretations. Int J Epidemiol 2021;49(6):2083-8.

18. Nunez Y, Gibson EA, Tanner EM, et al. Reflection on modern methods: good practices for applied statistical learning in epidemiology. Int J Epidemiol 2021;50(2):685-93.

19. Gunasekara FI, Richardson K, Carter K, Blakely T. Fixed effects analysis of repeated measures data. Int J Epidemiol 2014;43(1):264-9.

20. Bhaskaran K, Smeeth L. What is the difference between missing completely at random and missing at random? Int J Epidemiol 2014;43(4):1336-9.

21. Younge JO, Kouwenhoven-Pasmooij TA, Freak-Poli R, Roos-Hesselink JW, Hunink MM. Randomized study designs for lifestyle interventions: a tutorial. Int J Epidemiol 2015;44(6):2006-19.

22. Greenland S, Daniel R, Pearce N. Outcome modelling strategies in epidemiology: traditional methods and basic alternatives. Int J Epidemiol 2016;45(2):565-75.

23. Weinmayr G, Dreyhaupt J, Jaensch A, Forastiere F, Strachan DP. Multilevel regression modelling to investigate variation in disease prevalence across locations. Int J Epidemiol 2017;46(1):336-47.

24. Davies NM, Thomas KH, Taylor AE, et al. How to compare instrumental variable and conventional regression analyses using negative controls and bias plots. Int J Epidemiol 2017;46(6):2067-77.

25. Basagaña X, Pedersen M, Barrera-Gómez J, et al. Analysis of multicentre epidemiological studies contrasting fixed or random effects modelling and meta-analysis. Int J Epidemiol 2018;47(4):1343-54.

26. Lopez Bernal J, Cummins S, Gasparrini A. The use of controls in interrupted time series studies of public health interventions. Int J Epidemiol 2018;47(6):2082-93.

27. van Smeden M, Lash TL, Groenwold RHH. Reflection on modern methods: five myths about measurement error in epidemiological research. Int J Epidemiol 2020;49(1):338-47.

28. Rioux C, Lewin A, Odejimi OA, Little TD. Reflection on modern methods: planned missing data designs for epidemiological research. Int J Epidemiol. 2020;49(5):1702-11.

29. Bannick MS, McGaughey M, Flaxman AD. Ensemble modelling in descriptive epidemiology: burden of disease estimation. Int J Epidemiol 2021;49(6):2065-73.

30. Griswold ME, Talluri R, Zhu X, et al. Reflection on modern methods: shared-parameter models for longitudinal studies with missing data. Int J Epidemiol 2021;50(4):1384-93.

31. Sudharsanan N, Bijlsma MJ. Educational note: Causal decomposition of population health differences using Monte Carlo integration and the g-formula. Int J Epidemiol 2021;50(6):2098-107.

32. Duan C, Dragomir AD, Luta G, Breitling LP. Reflection on modern methods: understanding bias and data analytical strategies through DAG-based data simulations. Int J Epidemiol 2021;50(6):2091-7.

33. Thurber KA, Thandrayen J, Maddox R, et al. Reflection on modern methods: statistical, policy and ethical implications of using age-standardized health indicators to quantify inequities. Int J Epidemiol 2022;51(1):324-33.

34. Ross RK, Breskin A, Breger TL, Westreich D. Reflection on modern methods: combining weights for confounding and missing data. Int J Epidemiol 2021.

35. Hamra G, Maclehose R, Richardson D. Markov Chain Monte Carlo: an introduction for epidemiologists. Int J Epidemiol 2013;42(2):627-34.

36. Benjamin-Chung J, Arnold BF, Berger D, et al. Spillover effects in epidemiology: parameters, study designs and methodological considerations. Int J Epidemiol 2018;47(1):332-47.

37. Blakely T, Lynch J, Simons K, Bentley R, Rose S. Reflection on modern methods: when worlds collide-prediction, machine learning and causal inference. Int J Epidemiol 2021;49(6):2058-64.

38. Mittleman MA, Mostofsky E. Exchangeability in the case-crossover design. Int J Epidemiol 2014;43(5):1645-55.

39. Amorim LDAF, Cai J. Modelling recurrent events: a tutorial for analysis in epidemiology. Int J Epidemiol 2015;44(1):324-33.

40. Weinberg CR, Shi M, DeRoo LA, Basso O, Skjærven R. Season and preterm birth in Norway: A cautionary tale. Int J Epidemiol 2015;44(3):1068-78.

41. Westreich D, Edwards JK, Cole SR, Platt RW, Mumford SL, Schisterman EF. Imputation approaches for potential outcomes in causal inference. Int J Epidemiol 2015;44(5):1731-7.

42. Howe LD, Smith AD, Macdonald-Wallis C, et al. Relationship between mediation analysis and the structured life course approach. Int J Epidemiol 2016;45(4):1280-94.

43. Kim JH, Mooney SJ. The epidemiologic principles underlying traffic safety study designs. Int J Epidemiol 2016;45(5):1668-75.

44. Corbin M, Haslett S, Pearce N, Maule M, Greenland S. A comparison of sensitivity-specificity imputation, direct imputation and fully Bayesian analysis to adjust for exposure misclassification when validation data are unavailable. Int J Epidemiol 2017;46(3):1063-72.

45. Walker VM, Davey Smith G, Davies NM, Martin RM. Mendelian randomization: a novel approach for the prediction of adverse drug events and drug repurposing opportunities. Int J Epidemiol 2017;46(6):2078-89.

46. Harron KL, Doidge JC, Knight HE, et al. A guide to evaluating linkage quality for the analysis of linked data. Int J Epidemiol 2017;46(5):1699-710.

47. Leyrat C, Morgan KE, Leurent B, Kahan BC. Cluster randomized trials with a small number of clusters: which analyses should be used? Int J Epidemiol 2018;47(3):1012.

48. Lesko CR, Jacobson LP, Althoff KN, et al. Collaborative, pooled and harmonized study designs for epidemiologic research: challenges and opportunities. Int J Epidemiol 2018;47(2):654-68.

49. Morgan KE, Cook S, Leon DA, Frost C. Reflection on modern methods: calculating a sample size for a repeatability sub-study to correct for measurement error in a single continuous exposure. Int J Epidemiol 2019;48(5):1721-6.

50. Pearce N, Vandenbroucke JP. Educational note: types of causes. Int J Epidemiol 2020;49(2):676-85.

51. Hemming K, Taljaard M. Reflection on modern methods: when is a stepped-wedge cluster randomized trial a good study design choice? Int J Epidemiol 2020;49(3):1043-52.

52. Maringe C, Benitez Majano S, Exarchakou A, et al. Reflection on modern methods: trial emulation in the presence of immortal-time bias. Assessing the benefit of major surgery for elderly lung cancer patients using observational data. Int J Epidemiol 2020;49(5):1719-29.

53. Andrinopoulou E-R, Harhay MO, Ratcliffe SJ, Rizopoulos D. Reflection on modern methods: Dynamic prediction using joint models of longitudinal and time-to-event data. Int J Epidemiol 2021;50(5):1731-43.

54. Basagaña X, Barrera-Gómez J. Reflection on modern methods: visualizing the effects of collinearity in distributed lag models. Int J Epidemiol 2022;51(1):334-44.
